# Pixel-Level Segmented Longitudinal Optical Coherence Tomography Data of Macular Hole Surgery Outcomes

**DOI:** 10.1038/s41597-026-08154-7

**Published:** 2026-08-25

**Authors:** Yinzheng Zhao, Junjie Yang, Zhaosheng Li, Mathias Maier, Daniel Zapp, Peter Charbel Issa, Zhihao Zhao, Mohammad Ali Nasseri

**Affiliations:** 1 Klinik und Poliklinik für Augenheilkunde, Technische Universität München, München, Germany; 2 TUM School of Computation, Information and Technology (CIT), Technische Universität München, München, Germany; 3 Shunyi Women’s & Children’s Hospital of Beijing Children’s Hospital, Capital Medical University, Beijing, China; 4 Zhongshan Ophthalmic Center, Sun Yat-sen University, Guangzhou, China; 5 Department of Biomedical Engineering, University of Alberta, Alberta, Canada

## Abstract

Research involving idiopathic full-thickness macular hole (iFTMH) has been limited by a shortage of longitudinal, annotated public datasets. In this article, we introduce a longitudinal annotated dataset containing OCT scans from patients who previously had iFTMH surgery with multiple time points. The OCT dataset based on the publicly accessible cohort contains a total of 2,591 fovea-centered horizontal and vertical B-scans that were collected at one preoperative and six postoperative time points (from 2 weeks to 48 months) that allow researchers to examine retinal recovery. We provide high-quality, expert-validated, pixel-level segmentation masks for 12 retinal features, representing both pathological (e.g., macular hole, cysts, epiretinal membrane) and anatomical (e.g., external limiting membrane, ellipsoid zone, retinal pigment epithelium) structures. Technical validation revealed high inter- and intra-rater reliability for each annotation (e.g., >0.80 average Dice coefficient for selected features). We also provide a reference nnUNet baseline to facilitate future benchmarking and method comparison. Overall, this resource is intended to facilitate AI modeling, support quantitative studies of retinal healing, and verify new ophthalmic image analysis methods.

## Background & Summary

Idiopathic full-thickness macular hole (iFTMH) is a full-thickness neurosensory retinal defect at the fovea^1–4^, the central portion of the macula that is responsible for sharp, detailed vision^5–8^. This condition disrupts the neural retina, and results in symptoms of central vision loss, metamorphopsia, and central scotoma^9–11^. The incidence of iFTMH is estimated to be approximately 7.8 to 8.9 out of 100,000 individuals per year worldwide, with an increased incidence in females, and in individuals aged 60 years or older^12^. While the specific pathogenesis is still unknown, vitreomacular traction and anomalous posterior vitreous detachment are both believed to be significant risk factors^13–15^. Pars plana vitrectomy is the standard management for cases of iFTMH and is intended to relieve tractional forces, and promote hole closure, frequently resulting in improved anatomical and functional outcomes^16,17^.

The introduction of Optical Coherence Tomography (OCT) has transformed the treatment of idiopathic full-thickness macular hole (iFTMH). As a non-invasive imaging method, OCT generates cross-sectional images of the retinal layers with exceptional resolution^18^, ultimately allowing it to be accepted as the gold standard for the diagnosis, classification, and management of iFTMH. Preoperatively, OCT is used to characterize macular hole morphology^19^, and identify clinically relevant prognostic biomarkers^20^. Likewise, OCT has extremely high clinical value in regard to surgical planning, assessing anatomical closure and monitoring the restoration or changes in the microstructure of the retinal macular fovea over time^21–24^.

Shortcomings in public OCT datasets still limit data-driven research in postoperative iFTMH. There is a notable lack of longitudinal datasets that systematically follow patients from the preoperative period to postoperative recovery, all of which would be invaluable for modeling the dynamic morphological changes of the retina and understanding the mechanisms of healing. In addition, public datasets that have been annotated tended to contain only simple or sparse labels and often lacked the annotations of other clinically relevant structures. Public retinal OCT datasets such as RETOUCH^25^ and AROI^26^ have provided valuable benchmarks for fluid, layer, and retinal-structure segmentation. Nevertheless, disease-specific resources for postoperative idiopathic full-thickness macular hole remain limited, particularly datasets that combine longitudinal follow-up with dense pixel-level annotations of both pathological features and outer retinal recovery structures. This limits the development and validation of computational models focused on postoperative iFTMH healing.

In order to overcome these limitations and move the field forward, we introduce a new, longitudinal, and annotated OCT dataset of individuals who underwent iFTMH surgery. Our contribution includes high-quality, expert-validated, pixel-level segmentation labels of retinal structural features that span the breadth of pathological and healing patterns at the preoperative time point and multiple postoperative time points. This dataset will ultimately enable a wide range of applications, including the training and validation of artificial intelligence algorithms for automated image segmentation and quantitative analysis, quantitatively modelling disease progression and retinal recovery, the investigation of biomarkers, and serving as a benchmark for new image analysis methods.

## Methods

### Data Source and Cohort Description

The dataset utilized in this study is a publicly available, retrospective longitudinal cohort of patients who underwent surgery for iFTMH^27,28^. The data were originally collected and provided by Lachance^28^ *et al*. The dataset is openly accessible on the Kaggle^29^ platform. The input OCT images and clinical data are distributed under the GNU Lesser General Public License version 3.0. All data were fully anonymized to protect patient privacy before public release. The annotated dataset of this paper has been uploaded to Figshare^30^ in a compressed file format. The present Figshare^30^ dataset contains the newly generated pixel level segmentation masks, the organized image folder structure, and quality assessment summary.

The released clinical table contains 494 de-identified patient IDs. Each ID represents one de-identified patient and one operated eye. For patients with bilateral macular holes, only the first operated eye was included. No patient was assigned more than one ID. Of the 494 patient-level clinical records, 493 had at least one released OCT B-scan, whereas one record had no corresponding OCT image. Horizontal and vertical B-scans were paired when both orientations were available; missing counterparts were not imputed. The image archive contains 2,591 curated OCT B-scans with corresponding masks and quality records. The number of B-scans and IDs with available images at each time point is: baseline, 873 B-scans from 440 IDs; 2 weeks, 399 B-scans from 200 IDs; 3 months, 666 B-scans from 335 IDs; 6 months, 244 B-scans from 123 IDs; 12 months, 262 B-scans from 131 IDs; 24 months, 117 B-scans from 60 IDs; and 48 months, 30 B-scans from 15 IDs. The longitudinal data are therefore not completely paired across all time points. No ID has images available at all seven time points. The clinical table contains id, age, sex, pseudophakic status, macular-hole duration (mh_duration), elevated_edge, minimum macular-hole size (mh_size), and visual-acuity (VA) measurements at baseline, 2 weeks, 3 months, 6 months, and 12 months. Values were unavailable or unknown for 20 records in mh_duration, 21 records in elevated_edge, and 54 records in mh_size. Visual-acuity follow-up fields are incomplete. VA_2weeks is missing for 70 records, VA_3months for 133 records, VA_6months for 326 records, and VA_12months for 304 records. We have also added Supplementary Table S1 for availability and orientation completeness of OCT B-scans at each time point.

### Ground Truth Annotation

**Fig. 1:**
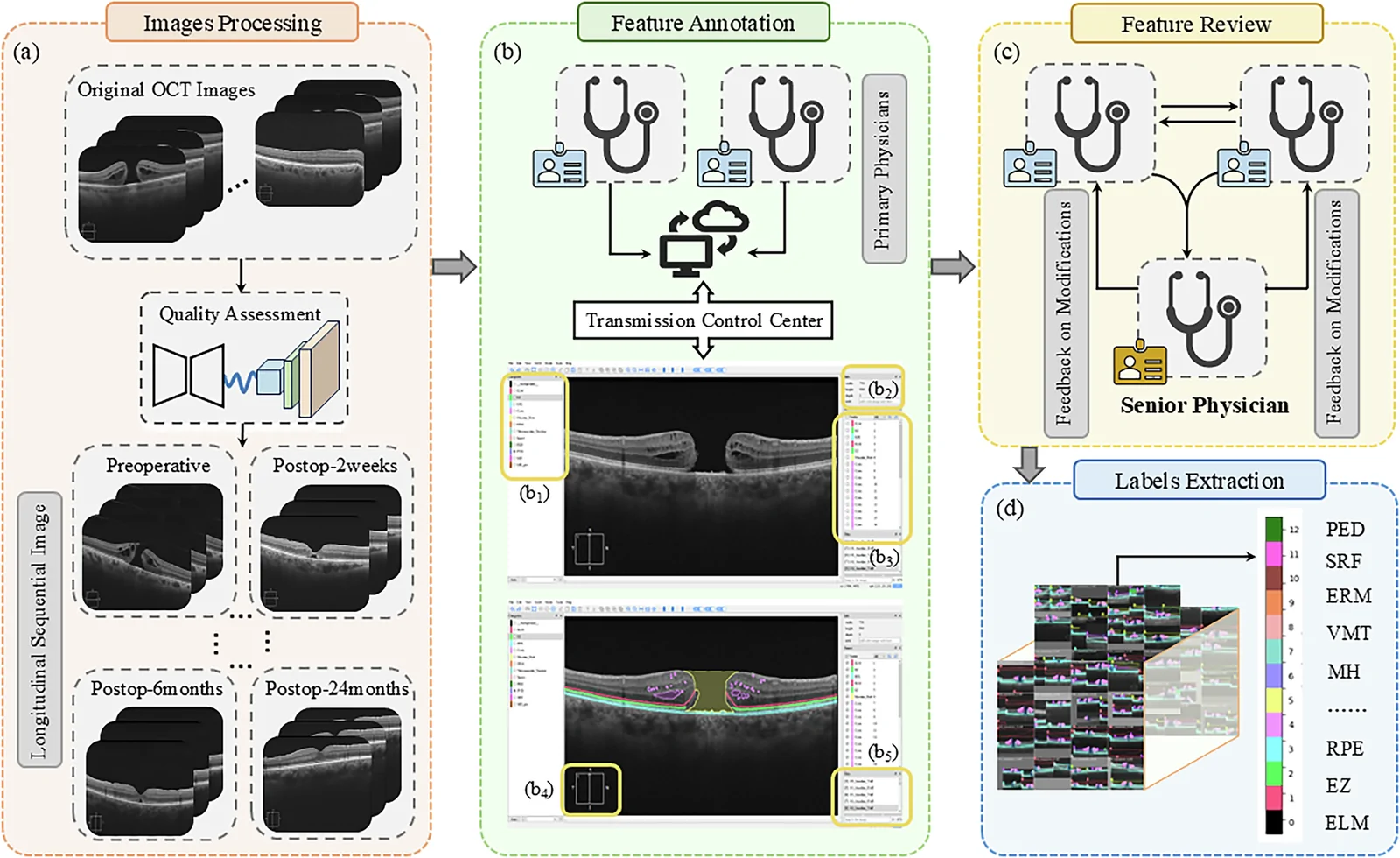
Flowchart of the research process. First, the raw dataset undergoes data preprocessing, with part (**a**) including data quality assessment and classification of the dataset based on postoperative follow-up time. Part (**b**) involves junior ophthalmologists using annotation software to label the classified OCT dataset and store the annotations on a device. The annotation software system includes components such as feature tags (b1), parameter windows (b2), annotation count windows (b3), graphical structure (b4), and image lists (b5). The annotation results from junior physicians are then compiled and evaluated with feedback from senior ophthalmologists (c), leading to the final determination of disease feature annotations (d).

Retinal feature annotation was performed according to a predefined multi-step protocol to ensure consistency and quality control. First, the curated OCT B-scan dataset, organized by patient identifier and time point, was imported into the ISAT image segmentation annotation platform (Intelligent Segmentation and Annotation, https://github.com/yatengLG/ISAT, Fig. 1). For each uploaded OCT image, annotators reviewed the existing labels and, when necessary, edited previous annotations, added or removed annotation classes, or assigned different color labels to distinguish multiple feature tags. Each OCT B-scan and its associated retinal feature annotations were assessed individually by independent annotators. After the initial annotation process, all B-scans were sequentially reviewed, revised, and confirmed by an attending vitreoretinal specialist with more than ten years of clinical and surgical experience. During this expert review, the specialist evaluated the quality and accuracy of each proposed annotation and requested reannotation or performed manual correction when needed, thereby ensuring the final validity of the retinal feature labels.

Masks are comprised of lines and points delineating the outer retinal boundaries. Region structures, including macular hole (MH), cysts, pigment epithelial detachment (PED), and sub-ERM space (Space) and subretinal fluid (SRF) were annotated as filled pixel regions. Thin reflective structures, including the external limiting membrane (ELM), ellipsoid zone (EZ), retinal pigment epithelium (RPE), epiretinal membrane (ERM), posterior vitreous detachment (PVD), and vitreomacular traction (VMT), were annotated as visible bands. Discontinuous but visible layer segments were annotated as separate segments. For all annotations, the start and end points of each label were determined by the first and last clearly visible OCT signal corresponding to that structure. Labels were not extended into regions where the anatomical boundary was obscured by shadowing, poor signal, motion artifacts, or fusion with adjacent layers. When a structure became discontinuous, each visible segment was annotated separately. Ambiguous start or end points were reviewed and finalized by the senior retinal specialist.

**Fig. 2:**
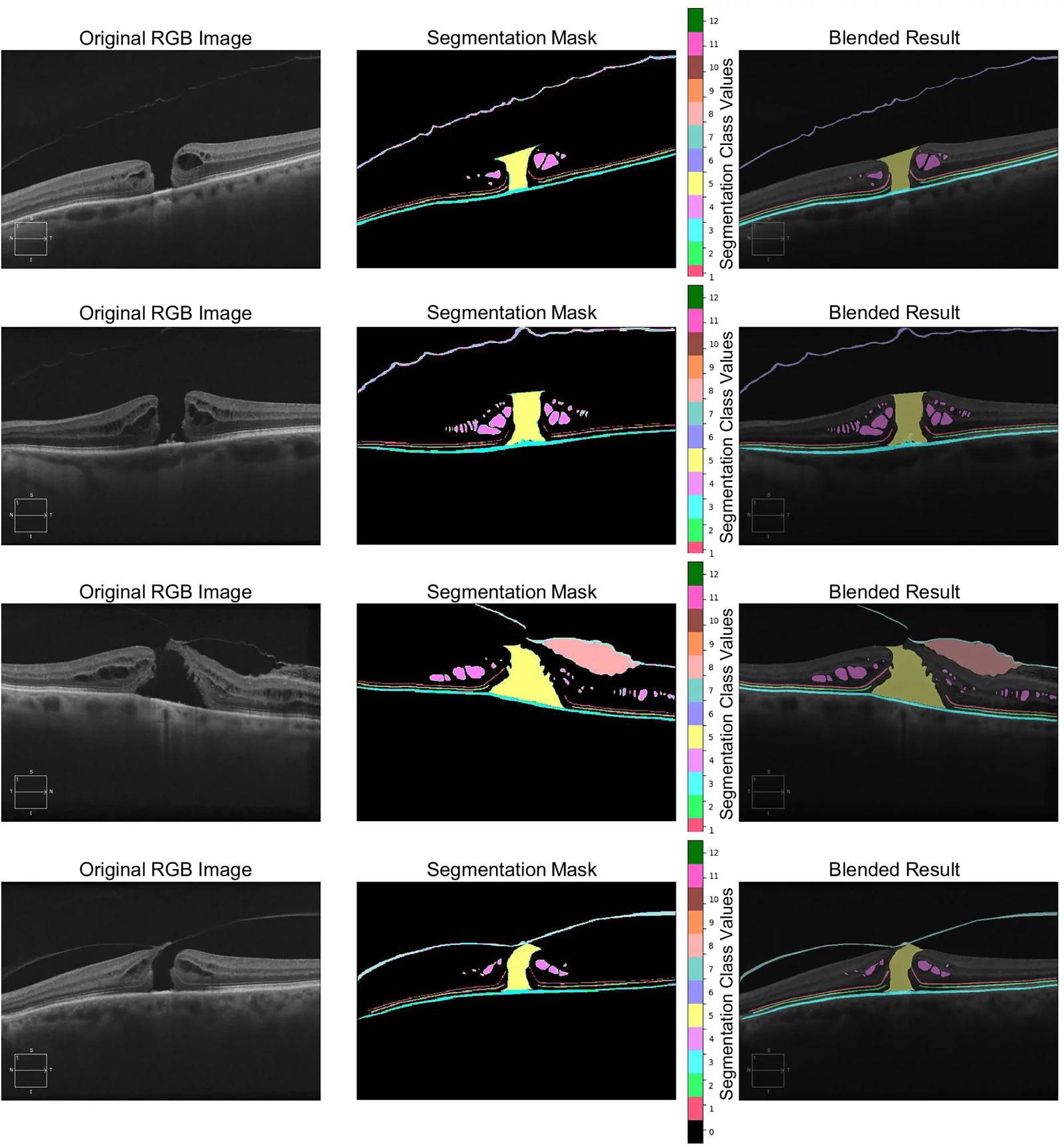
Representative OCT B-scans and corresponding segmentation masks illustrating the 12 annotated anatomical and pathological retinal features. Each class is displayed using a distinct color according to the class-index mapping shown in the legend. The definitive mapping is 0 Background, 1 ELM, 2 EZ, 3 RPE, 4 Cysts, 5 MH, 6 PVD, 7 VMT, 8 Space, 9 ERM, 10 MH Impending,11 SRF and 12 PED.

Our annotation scheme was designed to capture the comprehensive morphology of the iFTMH and associated pathologies. We defined a total of 12 distinct features for segmentation (Fig. 2). Each segmentation mask was stored as a single-channel PNG image, with each integer pixel value representing a specific annotated feature (Table 1). Value 0 denotes background, whereas values 1 to 12 correspond to ELM, EZ, RPE, Cysts, MH, PVD, VMT, Space, ERM, MH Impending, SRF, and PED, respectively. Because the released masks are single-channel class-index PNG files, each pixel was assigned to only one class. No automatic overwrites or priority rule was applied during mask generation. When two candidate labels appeared spatially close or potentially overlapping, the final pixel assignment was manually adjudicated by the senior vitreoretinal specialist according to the local OCT appearance and the predefined feature definitions. Pathological regions were limited to their visible boundaries, anatomical bands were labeled only where clearly visible, and pixels that could not be reliably assigned were labeled as background.

**Table 1:** Class-index mapping of single-channel segmentation masks.

Pixel Value	Class	Annotation Type
0	Background	Background
1	ELM	Visible band
2	EZ	Visible band
3	RPE	Visible band
4	Cysts	Filled region
5	MH	Filled region
6	PVD	Visible band
7	VMT	Visible band
8	Space	Filled region
9	ERM	Visible band
10	MH Impending	Filled region
11	SRF	Filled region
12	PED	Filled region

The protocol recognized that the number of pathological features is highest in the preoperative stage. Following surgical intervention, as the macular hole closes and the retina heals, the number of annotatable features progressively decreases. The definitions and annotation methods for all features are as follows:

MH: A full-thickness defect of the neurosensory retina at the fovea.

MH impending: A foveal depression or hyporeflective space indicating early-stage hole formation, characterized by outer retinal dehiscence while the inner retina remains intact.

Cysts: Round or oval hyporeflective spaces within the retinal layers, typically intraretinal edema.

ERM: Hyperreflective layer of tissue on the inner surface of the retina, often observed causing traction or retinal distortion. During annotation, only the visible membrane band was labeled.

Space: Hyporeflective gap between ERM and inner retinal surface. It was annotated as a filled region only when a clear separation between the ERM and retinal surface was visible. Focal spaces were annotated as separate filled regions, whereas ambiguous low-reflective areas without a clear ERM boundary were not labeled independently.

VMT: Persistent posterior vitreous attachment to the macular surface with visible tractional deformation of the foveal contour. The visible traction-related posterior vitreoretinal attachment band was annotated, rather than the entire deformed retinal area.

PVD: Complete separation of the posterior vitreous cortex from the retina, often visible as a detached hyperreflective line. The label followed the visible reflective band. We did not infer or extend the PVD label into regions where the posterior hyaloid was not visible.

ELM: A thin hyperreflective line demarcating the boundary between photoreceptor cell bodies and inner segments.

EZ: A distinct hyperreflective band representing the junction between the inner and outer photoreceptor segments, used as a marker of photoreceptor integrity. In regions of EZ disruption, the annotation stopped at the last clearly identifiable continuous EZ signal.

RPE: A strong, continuous hyperreflective layer beneath the neurosensory retina, critical for outer retinal health.

PED: Elevation of the RPE from the underlying Bruch’s membrane, forming a dome shaped detachment space. The lateral boundaries were placed where the elevated RPE returned to the adjacent baseline RPE contour. Ambiguous shallow elevations were reviewed by the senior specialist before final acceptance.

SRF: Located between the neurosensory retina and the RPE.

The set of annotated features was tailored to each clinical stage to reflect the evolving pathology. Table 2 provides a clear overview of the feature annotation scheme across the entire longitudinal timeline of the study, and the total pixel count for each class is provided in Supplementary Table S2.

**Table 2:** Number of B-scans containing each annotated retinal feature at each time point.

Feature Name	Preoperative	Postoperative 2weeks	Postoperative 3months	Postoperative 6months	Postoperative 12months	Postoperative 24months	Postoperative 48months	Total
ELM	873	399	666	244	262	117	30	2591
EZ	873	399	666	244	262	117	30	2591
RPE	873	399	666	244	262	117	30	2591
Cysts	849	21	22	4	14	20	4	934
MH	851	0	0	0	0	0	0	851
PVD	398	0	0	0	0	0	0	398
VMT	287	0	0	0	0	0	0	287
Space	95	0	0	0	0	0	0	95
ERM	467	7	1	1	2	4	0	482
MH Impending	40	0	0	0	0	0	0	40
SRF	2	0	0	0	0	0	0	2
PED	7	0	0	0	0	0	0	7
Image Count	873	399	666	244	262	117	30	2591

### Annotation Consistency Assessment

To quantitatively assess the reproducibility of the manual annotation protocol, a random subset of 100 OCT B-scans representing both preoperative and postoperative examinations was selected. For the inter-annotator agreement assessment, two junior physicians independently annotated the same 100 B-scans from scratch according to the predefined feature definitions, while blinded to each other’s annotations. Their masks were compared using the Dice similarity coefficient (DSC) and intersection over union (IoU). For the intra-annotator repeatability assessment, one of the two junior physicians independently re-annotated the same set of 100 OCT B-scans after a two-week washout period. During the repeat annotation session, the physician had no access to the initial annotations. Intra-annotator DSC and IoU were calculated by comparing this physician’s initial and repeated masks. For each retinal feature, DSC and IoU were calculated separately for each valid B-scan. A B-scan was considered valid for a given feature when that feature was present in at least one of the two masks being compared. B-scans in which the feature was absent from both masks were excluded from the class-specific analysis, whereas presence in only one mask resulted in DSC and IoU values of zero. The senior-specialist-adjudicated masks were not used as either member of the inter- or intra-annotator pairwise comparisons. Final ground-truth masks were produced after the consistency assessments by resolving discrepancies between the initial annotations through senior-specialist review and adjudication.

### Baseline Segmentation Model

To provide an optional reference benchmark for future users, we trained and evaluated a deep learning model for automated segmentation of the 9 retinal features. MH Impending (40 images), PED (7 images), and SRF (2 images) were excluded from the benchmark model due to limited data volume. Image-bearing patient IDs were assigned to a fixed patient-level split with target proportions of 70%/15%/15%, while balancing ID and B-scan distributions across time points. A random seed of 42 was used for reproducible tie-breaking. For model development, splitting was performed at the patient ID level. All horizontal and vertical B-scans, and all available visits, from the same ID were assigned to the same training, validation, or test subset. The baseline experiment is provided as a technical benchmark for mask usability and should not be interpreted as a clinical generalization study.

We provide a baseline model implementation to demonstrate the utility of our dataset for training segmentation models. We employed nnUNet^31^ for multi-class semantic segmentation of the 9 features we defined. SRF was not included because only two positive images were available, providing no positive sample in the validation or test subset. PED in 7 masks is retained in the complete annotation system to expand data coverage, but it is not included in the disease-specific baseline established in this study for iFTMH morphology and postoperative recovery. There are only 40 positive samples for MH impending, which is insufficient to support reliable model training and independent performance evaluation. During training, real-time data augmentation was applied to improve generalization. This included random horizontal flipping, rotation within ± 15°, scaling within ± 10%, and contrast adjustment. To reduce the effect of class imbalance, we used a combined loss function based on Focal Loss and Dice Loss. The trained model was evaluated on a held-out test set. Dice and IoU were used as the main evaluation metrics for each feature class. These results are provided only as reference benchmarks for future method comparison.

The baseline model employs the 2D full-resolution configuration of nnUNet v2. Training parameters include 512 × 512 image patches, a batch size of 16, and training for 250 epochs; the optimizer used is SGD (with Nesterov momentum of 0.99), with an initial learning rate of 1 × 10^−4^ (using polynomial decay) and a weight decay of 3 × 10^−5^.

## Data Records

The dataset is available at Figshare^30^. At the top level the archive contains “quality_assessment” directory, “clinical_data.csv” file and seven timepoint folders named baseline, 2weeks, 3months, 6months, 12months, 24months, and 48months. Each of these timepoint folders contains an OCT subfolder (< timepoint > _OCT) with B-scan images in TIFF format (.tiff) and a mask subfolder (< timepoint > _Masks) with disease feature segmentation results in PNG format (.png). Filenames follow the pattern < id > _ < timepoint > _ < orientation > , where orientation is H for horizontal B-scans or V for vertical B-scans. The “clinical_data.csv” file contains the columns id, age, sex, pseudophakic, mh_duration, elevated_edge, mh_size, visual acuity (VA) on five timepoints, and the reported values correspond to approximate ETDRS letter scores derived using 85-50 × log MAR.

The “quality_assessment” folder contains one CSV file per timepoint with the columns Filename, SNR (dB), SNR Quality, Laplacian Variance, Blur Status, and Signal Shield Detected. Segmentation masks are stored as single-channel class-index PNG files. Each image was converted to 8-bit grayscale after removal of padding and overlays. SNR was calculated as 20 log10 (μsignal/ σnoise), using the central 80% of the localized retinal band as the signal region and the vitreous strip immediately above it as the noise region; scans were categorized as Good (≥25 dB), Borderline (18–25 dB), or Poor (<18 dB). Blur was assessed using Laplacian variance, with values below 100 flagged as blurred, while signal shielding was flagged when a low-signal vertical band crossing the retina had a mean intensity <50% of adjacent retinal columns. Images were excluded only when the fovea-centered scan or the relevant retinal boundaries were not interpretable after review. Scans with borderline or poor SNR were retained when clinically annotatable.

## Technical Validation

### Dataset Characteristics

The dataset contains 2,591 OCT images along with their corresponding Ground Truth images. Across these images, SNR Quality was Good in 1,249 images, Borderline in 1,014 images, and Poor in 328 images. All retained images were classified as not blurry, and 287 images were flagged for signal shielding. Most examinations contained fovea-centered horizontal and vertical B-scans. However, both orientations were not available for every visit, and the morphological presentation of the disease changes over time with follow-up. Table 2 summarized the disease state changes. Some disease features completely disappeared two weeks after surgery, while a few persist up to two years post-surgery, and the intrinsic anatomical structure of the retinal layers remained intact. Table 3 summarized the clinical data volumes, including 155 males and 339 females. The average patient age was 67.84 years with a standard deviation of 8.00 years. The total number of patients varies across different follow-up stages. The numerical results are calculated using the number of patients in the corresponding stage as the denominator.

**Table 3:** Summary of clinical data characteristics.

Characteristics	Values	Total
Age	67.84 ± 8.00	494
Gender		
Male	155	155
Female	339	339
VA		
Preoperative	51.17 ± 14.77	494
Postoperative 2 W	58.05 ± 18.40	424
Postoperative 3 M	65.88 ± 12.31	361
Postoperative 6 M	66.33 ± 11.58	168
Postoperative 12 M	67.63 ± 11.98	190

**Fig. 3:**
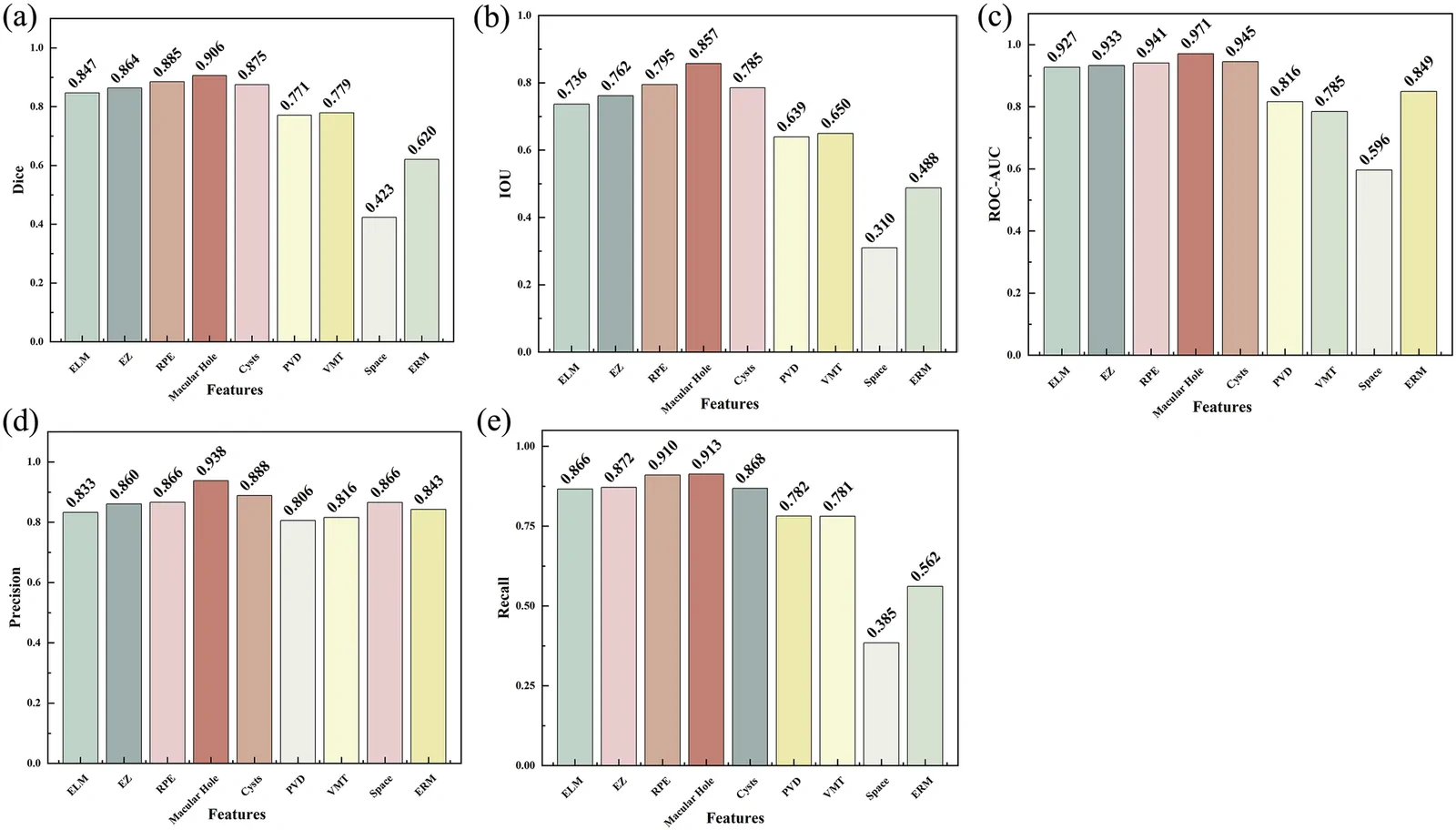
Optional reference nnUNet baseline performance on the held-out test subset (a-e). Panels show class-specific Dice (**a**), IoU (**b**), ROC-AUC (**c**), Precision (**d**), and Recall (**e**) for the nine retinal features included in the reference benchmark.

As an additional reference for data users, we evaluated an nnUNet model on the held-out test subset using standard pixel-level segmentation metrics. Detailed class-specific results are presented in the Usage Notes and Fig. 3. This experiment is provided as a reference benchmark for future method development and comparison.

### Annotation Consistency Assessment

To verify the robustness and reproducibility of our annotation protocol, we conducted rigorous inter-annotator and intra-annotator reliability analyses. Inter- and intra-rater Dice/IoU scores were used to quantify the reproducibility of the preliminary manual annotation protocol. The final ground-truth masks were produced through senior-specialist adjudication before any baseline model training or evaluation.

**Fig. 4:**
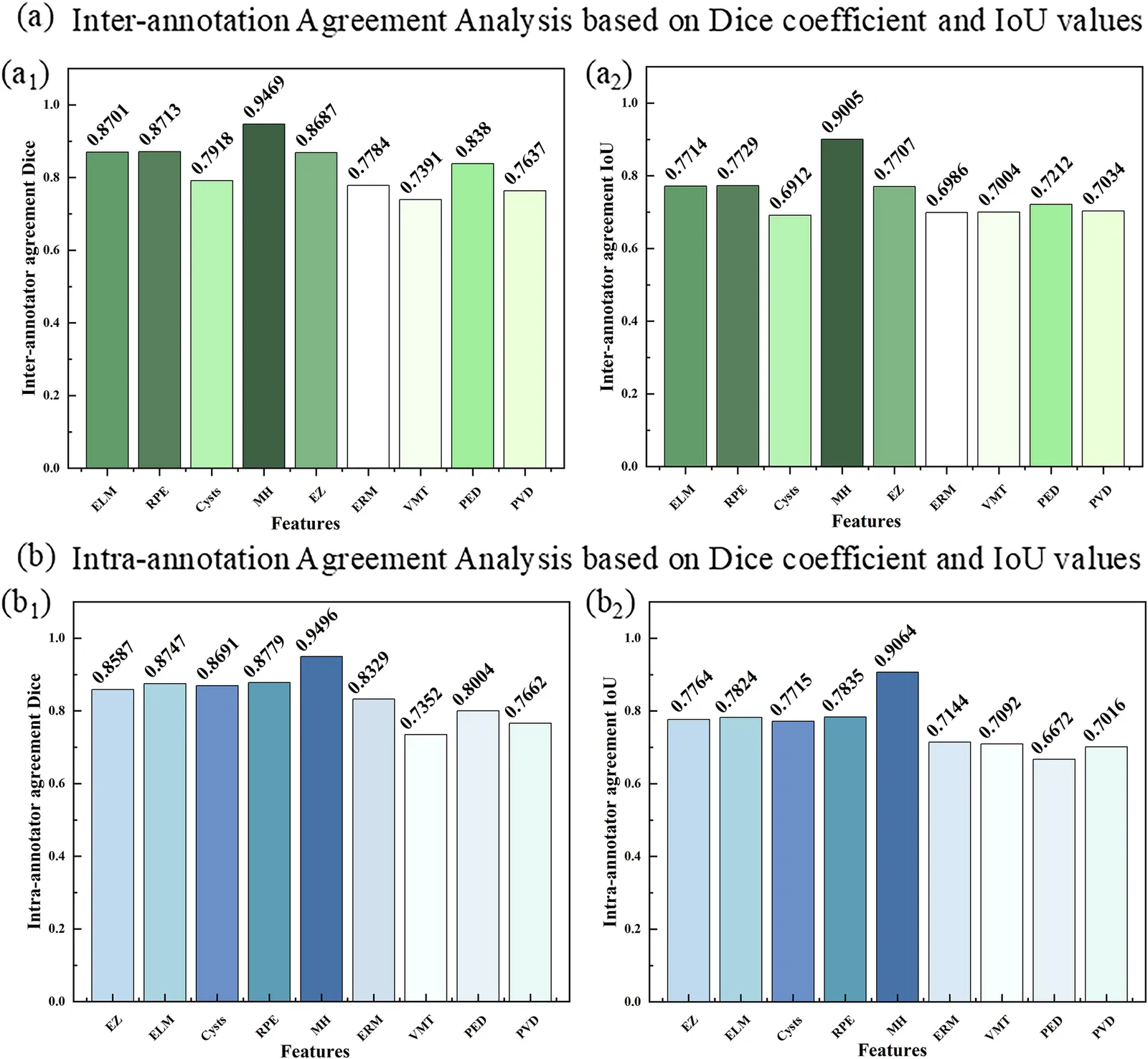
Annotation consistency evaluation. (**a**) Inter-annotator consistency analysis. The average Dice (a_1_) coefficient was above 80% for major features. The average IoU (a_2_) was above 70% for most features. (**b**) Intra-annotator consistency analysis. The average Dice (b_1_) coefficient and IoU (b_2_) for major features were both above 70%.

The evaluation results (Fig. 4) quantify the consistency level of our annotation process. Inter-annotator reliability assessed the consistency between two different junior physicians annotating the same set of images at the same time point (Fig. 4a). The analysis results showed high consistency between the two annotators for anatomical structures and pathological features with clear morphology and well-defined boundaries. For example, the average Dice coefficients for the ELM, EZ, RPE, and MH all exceeded 0.80, with corresponding high IoU values. This indicates that our definitions of these key features are clear and unambiguous. Intra-annotator reliability assessed the consistency of the same junior physician when repeatedly annotating the same image set at different time points (Fig. 4b). The results showed very high consistency within the annotator. For most features, the intra-annotator Dice coefficients were generally higher than or equal to the inter-annotator Dice coefficients. Particularly for MH, the Dice coefficient reached 0.95. Even for more challenging features, such as PVD (approximately 0.77) and ERM (0.83), intra-annotator consistency remained strong, indicating that the annotator could consistently follow the established annotation criteria. We have also added Supplementary Table S3 for inter- and intra-annotator agreement for OCT feature annotations, Supplementary Table S4 for distribution of annotated features in the 100 images selected.

These two analyses indicate that our annotation protocol is robust and reliable. For key, structured pathological features, the annotations exhibit high reproducibility. Although there are some interpretive differences in fine structures, these discrepancies were resolved and unified through the final review and arbitration process by senior experts, ensuring the overall high quality and accuracy of our dataset.

## Usage Notes

This dataset provides a longitudinal, annotated OCT resource for investigating the pathophysiology of iFTMHs and postoperative retinal recovery. It may support the development and benchmarking of AI-based segmentation algorithms, longitudinal analyses of macular hole closure and retinal layer restoration, and studies of prognostic imaging biomarkers linked to visual outcomes using the accompanying clinical data. Users should consider the dataset structure, including horizontal and vertical B-scans, as well as class imbalance in pathological features across postoperative time points. Appropriate strategies should therefore be adopted during model training and analysis. We encourage researchers to use this resource for ophthalmic image analysis and clinical research and to cite this paper when using the dataset.

A reference nnUNet model was evaluated on the held-out test subset using the Dice similarity coefficient, IoU, area under the receiver operating characteristic curve (ROC-AUC), precision, and recall (Fig. 3). The model achieved Dice coefficients of 0.89, 0.86, and 0.85 for the RPE, EZ, and ELM, respectively. For the principal pathological features, the Dice coefficients were 0.91 for MH and 0.88 for cysts. PVD achieved a Dice coefficient of 0.77. Lower Dice and IoU values were observed for smaller or less frequently represented traction-related features, including VMT, ERM, and Space, although their precision values were 0.82, 0.84, and 0.87, respectively. These results are provided solely as optional reference benchmarks for method comparison. The differences should be considered when selecting sampling strategies, loss functions, and evaluation procedures, particularly for rare or spatially small classes.

This dataset can support exploratory analyses linking segmented OCT morphology with available visual-acuity outcomes. However, it should not be treated as a complete surgical prognostic registry. Hole stage is not provided as a separate clinical-table variable. Baseline outer retinal status is not provided as a spreadsheet variable but can be assessed from the ELM, EZ, and RPE segmentation masks. Individual-level surgical details, including surgical-procedure variations, ILM peeling status, tamponade material, and dye use, are not available in the released public clinical table and should not be inferred.

## Supplementary information

Supplementary information

## Data Availability

The dataset generated in this study is available at Figshare^30^.

## Code availability

No custom code was developed specifically for generating the released dataset.
