# Supplementary material for: Pixel-Level Segmented Longitudinal Optical Coherence Tomography Data of Macular Hole Surgery Outcomes
Source: Sci Data. 2026 Aug 25;13:1230. doi: 10.1038/s41597-026-08154-7 (PMC13507111; doi:10.1038/s41597-026-08154-7)
Supplement: Supplementary file 1 [file 41597_2026_8154_MOESM1_ESM.pdf]

## Supplementary Tables

**Supplementary Table S1. Availability and orientation completeness of OCT B-scans at each time point**

| Time point | Available IDs | Missing IDs, n (%) | B-scans | Paired H/V IDs, n (%) |
|---|---|---|---|---|
| Baseline | 440 | 54 (10.9%) | 873 | 433 (98.4%) |
| 2 weeks | 200 | 294 (59.5%) | 399 | 199 (99.5%) |
| 3 months | 335 | 159 (32.2%) | 666 | 331 (98.8%) |
| 6 months | 123 | 371 (75.1%) | 244 | 121 (98.4%) |
| 12 months | 131 | 363 (73.5%) | 262 | 131 (100.0%) |
| 24 months | 60 | 434 (87.9%) | 117 | 57 (95.0%) |
| 48 months | 15 | 479 (97.0%) | 30 | 15 (100.0%) |

Missing-ID percentages were calculated using the 494 patient-level clinical records as the denominator. One patient had no released OCT image at any time point. Paired H/V scans indicate that both horizontal and vertical B-scans were available for the same ID at the corresponding time point.

**Supplementary Table S2. Total number of masks and total pixel values for each category**

| Pixel Value | Class | Image Counts | Total number of pixels |
|---|---|---|---|
| 0 | Background | 2591 | -- |
| 1 | ELM | 2591 | 6449406 |
| 2 | EZ | 2591 | 6295361 |
| 3 | RPE | 2591 | 10893450 |
| 4 | Cysts | 934 | 5272186 |
| 5 | MH | 851 | 2307989 |
| 6 | PVD | 398 | 1199910 |
| 7 | VMT | 287 | 756132 |
| 8 | Space | 95 | 42740 |
| 9 | ERM | 482 | 343424 |
| 10 | MH Impending | 40 | 58305 |
| 11 | SRF | 2 | 796 |
| 12 | PED | 7 | 1320 |

Image counts represent the number of images in which each category was present, while total pixel values represent the cumulative number of annotated pixels for that category across the dataset.

**Supplementary Table S3. Inter- and intra-annotator agreement for OCT feature annotations.**

| Feature | Inter-annotator Dice | Inter-annotator IoU | Intra-annotator Dice | Intra-annotator IoU |
|---|---|---|---|---|
| ELM | 0.8701 ± 0.0302 | 0.7714 ± 0.0495 | 0.8747 ± 0.0225 | 0.7824 ± 0.0349 |
| RPE | 0.8713 ± 0.0253 | 0.7729 ± 0.0391 | 0.8779 ± 0.0271 | 0.7835 ± 0.0430 |
| Cysts | 0.7918 ± 0.2156 | 0.6912 ± 0.2045 | 0.8691 ± 0.0471 | 0.7715 ± 0.0726 |
| MH | 0.9469 ± 0.0286 | 0.9005 ± 0.0500 | 0.9496 ± 0.0452 | 0.9064 ± 0.0745 |
| EZ | 0.8687 ± 0.0487 | 0.7707 ± 0.0650 | 0.8587 ± 0.0589 | 0.7764 ± 0.0781 |
| ERM | 0.7784 ± 0.0916 | 0.6986 ± 0.2278 | 0.8329 ± 0.0248 | 0.7144 ± 0.0362 |
| VMT | 0.7391 ± 0.0822 | 0.7004 ± 0.0230 | 0.7352 ± 0.0809 | 0.7092 ± 0.0935 |
| PED | 0.8380 ± 0.0000 | 0.7212 ± 0.0000 | 0.8004 ± 0.0000 | 0.6672 ± 0.0000 |
| PVD | 0.7637 ± 0.0343 | 0.7034 ± 0.0879 | 0.7662 ± 0.0341 | 0.7016 ± 0.0983 |

Values are presented as mean ± standard deviation (SD). Inter-annotator agreement was assessed between different annotators evaluating the same images, whereas intra-annotator agreement was assessed from repeated annotations of the same images by the same annotator at different time points.

**Supplementary Table S4. Distribution of annotated features in the 100 images selected for inter- and intra-annotator agreement assessment**

| Feature | Annotator A | Annotator A1 | Annotator B |
|---|---|---|---|
| ELM | 100 | 100 | 100 |
| EZ | 100 | 100 | 100 |
| RPE | 100 | 100 | 100 |
| Cysts | 33 | 33 | 31 |
| MH | 25 | 25 | 25 |
| ERM | 10 | 9 | 8 |
| PVD | 10 | 9 | 9 |
| VMT | 4 | 6 | 6 |
| PED | 1 | 1 | 1 |
| Space | 0 | 0 | 0 |
| MH Impending | 0 | 0 | 0 |
| SRF | 0 | 0 | 0 |

Numbers indicate the number of images in which each feature was annotated as present. Multiple features could coexist in the same image; therefore, the counts across features do not sum to 100.
